# Supplementary material for: Reconciling Mining with the Conservation of Cave Biodiversity: A Quantitative Baseline to Help Establish Conservation Priorities
Source: PLoS One. 2016 Dec 20;11(12):e0168348. doi: 10.1371/journal.pone.0168348 (PMC5173368; doi:10.1371/journal.pone.0168348)
Supplement: S1 Dataset — (ZIP) [file pone.0168348.s002.zip › Taxa/Serra Norte/SN_2013/N4WS/Lista N4WS-68.pdf]

CAVIDADE N4WS-0068

Classe	Ordem	Fam/Outros	Gên/Outros	Espécie	S-U	U-S	Zon.
Arachnida	Acari	Trombiculidae		sp.1	X		E
Arachnida	Araneae	Pholcidae	*Leptopholcus*	sp.1	X	X	E
Arachnida	Araneae	Pholcidae	*Mesabolivar*	*eberhard*	X	X	E
Arachnida	Araneae	Symphytognathidae	*Anapistula*	jovem	X		E
Arachnida	Araneae	Theridiidae	*Achaearanea*	sp.1	X		E
Arachnida	Araneae	Theridiosomatidae	*Plato*	sp.1	X	X	E
Entognatha	Collembola	Isotomidae		sp.1		X	E
Entognatha	Collembola	Paronellidae		sp.4	X		E
Insecta	Coleoptera	Carabidae		sp.13		X	E
Insecta	Diptera	Cecidomyiidae	Cecidomyiinae	sp.	X		E
Insecta	Diptera	Chaoboridae		sp.		X	E
Insecta	Diptera	Chironomidae		sp.	X		E
Insecta	Diptera	Culicidae	*Culex*	sp.		X	E
Insecta	Diptera	Psychodidae	*Sciopemyia*	*sordellii*	X	X	E
Insecta	Diptera	Tipulidae	Tipulinae	sp.	X		E
Insecta	Homoptera	Cixiidae		jovem		X	E
Insecta	Hymenoptera	Formicidae	*Crematogaster*	sp.1	X		E
Insecta	Lepidoptera	Noctuoidea		sp.1	1	7	E
Insecta	Lepidoptera	Noctuoidea		sp.2	X	X	E
Insecta	Orthoptera	Phalangopsidae	*Phalangopsis*	sp.		7	E
Insecta	Psocoptera	Amphipsocidae	*Monocladellus*	sp.1	X		E
Insecta	Psocoptera	Psyllipsocidae	*Psyllipsocus*	sp.2	X		E
Malacostraca	Isopoda	Scleropactidae	*Circoniscus*	*buckupi*	X		E
Turbellaria	Tricladida			sp.1		X	E
Amphibia	Anura	Strabomantidae	*Pristimantis*	cf. *fenestratus*		1	E
Mammalia	Chiroptera	Emballonuridae	*Peropteryx*	sp.	1	1	E
